# Application of positron emission tomography in psychiatry—methodological developments and future directions

**DOI:** 10.1038/s41398-022-01990-2

**Published:** 2022-06-14

**Authors:** Simon Cervenka, Andreas Frick, Robert Bodén, Mark Lubberink

**Affiliations:** 1 Department of Medical Sciences, Psychiatry, Uppsala University, Uppsala, Sweden; 2 Centre for Psychiatry Research, Department of Clinical Neuroscience, Karolinska Institutet and Stockholm Health Care Services, Region Stockholm, Stockholm, Sweden; 3 Department of Surgical Sciences, Uppsala University, Uppsala, Sweden

**Keywords:** Biomarkers, Neuroscience

## Abstract

Mental disorders represent an increasing source of disability and high costs for societies globally. Molecular imaging techniques such as positron emission tomography (PET) represent powerful tools with the potential to advance knowledge regarding disease mechanisms, allowing the development of new treatment approaches. Thus far, most PET research on pathophysiology in psychiatric disorders has focused on the monoaminergic neurotransmission systems, and although a series of discoveries have been made, the results have not led to any material changes in clinical practice. We outline areas of methodological development that can address some of the important obstacles to fruitful progress. First, we point towards new radioligands and targets that can lead to the identification of processes upstream, or parallel to disturbances in monoaminergic systems. Second, we describe the development of new methods of PET data quantification and PET systems that may facilitate research in psychiatric populations. Third, we review the application of multimodal imaging that can link molecular imaging data to other aspects of brain function, thus deepening our understanding of disease processes. Fourth, we highlight the need to develop imaging study protocols to include longitudinal and interventional paradigms, as well as frameworks to assess dimensional symptoms such that the field can move beyond cross-sectional studies within current diagnostic boundaries. Particular effort should be paid to include also the most severely ill patients. Finally, we discuss the importance of harmonizing data collection and promoting data sharing to reach the desired sample sizes needed to fully capture the phenotype of psychiatric conditions.

## Background

In a given year, one out of six individuals in Europe will suffer from a mental health problem, and it is estimated that the total cost to European societies for these disorders is 600 billion euros [1]. Recovery rates for schizophrenia have not improved for several decades [2], and similarly the global disease burden of depressive and anxiety disorders, both ranked among the top 25 leading causes of burden, has not diminished since 1990 [3]. Importantly, the identification of new treatment approaches that could improve this situation is hampered by a lack of understanding of the pathophysiology of mental disorders. Molecular imaging methods such as positron emission tomography (PET) offer a unique opportunity to measure brain biochemical markers in vivo, and have since their advent in the 1980:s been considered a key approach to advance the field.

Psychiatry was indeed an early focus in the field of quantitative PET, with the very first clinical studies being conducted in patients with schizophrenia. Demonstrations of the antipsychotic mechanism of action using dopamine D2 receptor (D2-R) radioligands such as [^11^C]raclopride was followed by a series of occupancy studies outlining thresholds for treatment efficacy and side effects [4, 5]. Arguably, the resulting change in dosing regimens remains the most significant contribution of PET research to clinical psychiatry to date. Since then, a wealth of studies have attempted to investigate the pathophysiology of psychiatric disorders, mainly based on disease models involving monoamine neurotransmission systems. To name a few examples, studies of dopaminergic function in psychosis and schizophrenia have collectively shown increased dopamine synthesis and release capacity as indexed by increased [^18^F]DOPA uptake and more pronounced amphetamine-induced decreases in D2-R receptor binding, a small increase in striatal D2-R availability and lower D2-R availability in the thalamus [6, 7]. In major depressive disorder (MDD), findings point to reduced serotonin transporter [8] and serotonin 1A receptor (5HT1A-R) availability [9], whereas in patients with anxiety disorders the evidence suggests decreases in 5HT1A and benzodiazepine receptors [10]. Several new radioligands for monoaminergic neurotransmission targets have been developed in recent years, such as tracers with increased sensitivity for endogenous neurotransmitter levels [11–14], that may further advance our understanding of the involvement of these systems. However, thus far this line of research has not led to new diagnostic tools or treatments for clinical use, prompting research based on new models of disease pathophysiology.

In order to advance PET research to the point of improving the care of psychiatric patients, some important challenges remain. First, there is a need to identify disease-related processes that act upstream or parallel to the observed abnormalities in monoaminergic dysfunction. To accomplish this, radioligands for new targets need to be developed, and a wider methodological scope is required to outline molecular pathways from genetics and environmental factors to the dysfunctional information processing that underlies psychiatric symptoms. Second, psychiatric populations are characterized by considerable heterogeneity in clinical phenotype—likely corresponding to heterogeneity in pathophysiological mechanisms. It is generally acknowledged that current diagnostic boundaries are ill-matched to the underlying biology, presenting a critical challenge to advancing knowledge [15]. Third, psychiatric patients are a vulnerable group, calling for the simplification of clinical research protocols. The most severely ill patients are typically not included in research studies at all, owing to difficulties in adhering to cumbersome study procedures or not being able to provide informed consent, limiting our understanding of the full phenotype associated with mental disorders.

In this review, we aim to describe recent advancements in methodological development in PET that may address these challenges, and to what extent they have been applied in clinical psychiatric research.

### New targets and radioligands

A key bottleneck in clinical PET research in general is the availability of radioligands for targets of interest. In the following, we highlight areas of development with specific relevance to psychiatric disorders.

### Intracellular targets

One approach to go beyond the level of neurotransmission signalling is to target intracellular enzymes in neurons [16–19]. This approach allows for investigating the activation state of groups of neurons during a certain condition, and may also elucidate if a dysfunction of that specific intracellular mechanism is part of the underlying pathophysiology of specific disorders [20]. Examples of this strategy are studies of the cyclic nucleotide phosphodiesterase (PDE) family of enzymes, which metabolize cyclic nucleotides and thereby regulate the signalling of these second messenger systems. PDE10A has been of recent interest in schizophrenia research [17,18]. In the brain, PDE10A is highly localized to the striatum, where it regulates the output of the direct and indirect striatal pathways that are implicated in several psychiatric conditions. PDE10A radioligands have thus far only been used in small samples of schizophrenia patients, showing inconclusive results [17,18].

Another enzyme in the PDE family, PDE4, is the main enzyme in the brain responsible for metabolizing 3′,5′-monophosphate (cAMP) into its inactive state. The cAMP signalling pathway is a major second messenger pathway of G-coupled receptors, involved in a range of intracellular processes including propagating neuron-neuron signals, and is hence of high relevance for many psychiatric conditions. The radioligand [^11^C]rolipram, a nonselective PDE4 antagonist, was used to show reduced cAMP activity in MDD compared to controls, and an upregulation after selective serotonin reuptake inhibitor (SSRI) treatment [19]. Indeed, PDE4 inhibitors have been suggested as a treatment in psychiatric conditions based on positive effects on neuroplasticity and neuroinflammation, but adverse effects hamper their implementation [21]. More selective targeting of PDE4 subtypes (i.e. PDE4 A/B/C/D) and increased understanding of the regulation of conformational states may be keys to overcome the adverse effects. In this respect, recent efforts have been made to develop radioligands with higher specificity for certain PDE4 subtypes [22], but these have yet to be applied in MDD or other psychiatric conditions.

### Immune markers

Genetic and epidemiologic data support a role for the immune system in psychotic disorders, depression and anxiety disorders as well as neurodevelopmental disorders. During the last decade, intensive efforts have aimed to identify PET markers for dysregulated immune function which for instance could be used to stratify patients for treatments targeting the immune system. To date, the most established method is to target the 18kD translocator protein (TSPO), which is expressed in activated microglia and astrocytes. Whereas initial studies in schizophrenia using the first generation TSPO radioligand [^11^C]PK-11195 showed increased binding in patients, this could not be confirmed in ensuing studies using TSPO radioligands with increased sensitivity such as [^11^C]PBR28 and [^18^F]FEPPA. Instead, data has converged towards lower binding across several brain regions [23, 24]. In contrast, six out of seven TSPO studies in MDD have shown increased binding compared with controls [24].

TSPO is not specific to microglia [25, 26], and in vitro studies have shown conflicting results regarding the specificity of TSPO for pro-inflammatory cells [27, 28]. There are currently several radioligands under investigation to find more specific markers for neuroinflammatory processes. Radioligands such as [^11^C]deprenyl and [¹¹C]SL25.1188, targeting the enzyme MAO-B, have been considered markers of astroglial activation. The latter radioligand has been used to show increased binding in the prefrontal cortex in patients with MDD [29], whereas a small study found no difference in binding in post-traumatic stress disorder (PTSD) patients [30]. With regard to microglial and proinflammatory markers, some promising candidates have been developed for Cox-1 and Cox-2, CSF1R and P2X7 [24], but to our knowledge, none of these have so farbeen used to study psychiatric disorders.

#### Synaptic density

**Fig. 1:**
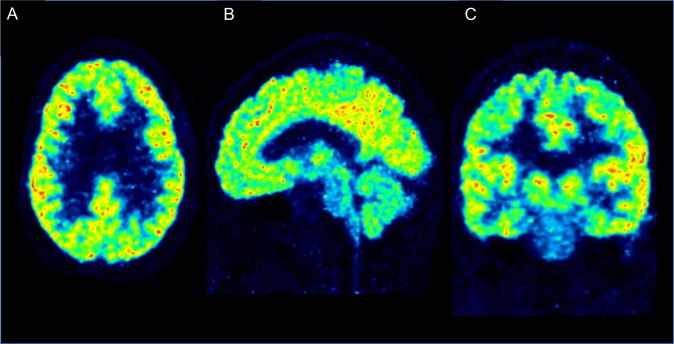
PET SV2A in early stage psychosis. An image of mean [^11^C]UCB-J uptake across frames in a first episode psychosis patient, horizontal (**A**), sagittal (**B**) and coronal (**C**) projections. Courtesy of Karolinska Institutet PET Centre.

A wealth of literature supports aberrant connectivity in several psychiatric conditions, as indicated indirectly using functional magnetic resonance imaging (MRI) modalities and post-mortem studies. In order to investigate connectivity at the molecular level, there has been an increasing interest in identifying targets for synaptic density. The PET radioligand [^11^C]UCB-J binds to the synaptic vesicle glycoprotein 2A (SV2A), which has shown high correspondence to the established post-mortem marker synaptophysin in non-human primate studies [31]. [^11^C]UCB-J has been applied in patients with MDD and/or PTSD, showing an inverse correlation between depressive symptoms and SV2A density, as well as an association between SV2A density and functional connectivity in prefrontal cortex as measured using resting-state functional MR (fMRI) [32]. The radioligand has been used in medicated patients with chronic schizophrenia, showing decreases in SV2A binding across several cortical regions [33, 34]. Recently, a small study found decreased SV2A binding in the hippocampus in users of cannabis [35], a drug considered to be a major risk factor for schizophrenia [36]. An important aim following the observations in MDD and schizophrenia is to investigate if changes in synaptic density are present already in the early stages of psychiatric disorders (Fig. 1).

#### Future developments

PET neuroreceptor ligands are generally based on small, lipophilic molecules that can easily cross the blood-brain barrier (BBB). In oncological PET, the use of labelled antibodies with high specificity and affinity has long been of interest. To enable brain uptake sufficient for imaging, recent developments have shown the possibility of designing bi-specific antibodies that undergo active transport across the BBB by transferrin-receptor-mediated transcytosis visualizing, e.g., amyloid-beta [37]. Although these methods have only been demonstrated in small-animal studies at this point [38] and have been developed primarily for application in neurodegenerative disorders, there could be potential applications in psychiatry given the interest in immune-related disease mechanisms. A limitation with labelled antibodies is their slow clearance from the blood which may require long-lived isotopes such as ^79^Zr for labelling, resulting in high radiation doses. A novel approach to overcome this is so-called pre-targeting using bioorthogonal chemistry, i.e., administration of an intermediate compound such as an antibody that binds to the target of interest, which can in turn be targeted by small molecules labelled with short-lived PET isotopes after the circulating antibody has cleared [39]. Hence, the antibody is labelled after it has bound to its target in the body and cleared from the blood, improving image contrast and reducing radiation dose compared to direct imaging of labelled antibodies. A challenge with this approach that will need to be solved is how to measure target availability, as the PET signal will be dependent on the kinetics of both the antibody and the tracer. If applied to psychiatry, his approach could potentially also be used to increase the ecological validity of studies in conditions with a fluctuating course, i.e., by administering the pre-targeting compound immediately when patients are experiencing symptoms, allowing for PET scanning to be done at a later time.

#### Radioligand validation and availability

When exploring new radioligands for use in psychiatry, it should be kept in mind that PET tracers are typically designed to fulfil a set of specific imaging properties [40]. The development involves validating the specificity of the tracer for its target using blocking or displacement studies, identifying the optimal method for quantitative analysis by studies on tracer kinetic analysis, and establishing the precision of binding measures using test-retest studies. However, the fact that a tracer has been validated for one application does not necessarily mean that it can be utilized for other applications. For instance, a utility of the medium affinity D2-R radioligand [^11^C]raclopride, which has primarily been used to investigate high-density striatal regions, has been suggested also for measurements in low-density cortical brain regions based on test-retest reliability studies and between-sample comparisons. Importantly, competition studies and within-subject comparisons to high-affinity radioligands showed low validity for this approach [41, 42]. Similarly, high image quality and accurate quantification of binding potential during the time typically deemed practical for dynamic PET scans require preferably a high extraction, a relatively short time to (transient) equilibrium, and fast clearance of the tracer. These characteristics may not be optimal for steady-state PET/fMRI protocols aiming to quantify neurotransmitter release with the high temporal resolution, where both a fast association and dissociation from the target as well as a fast clearance of non-bound tracer from the brain are ideally required.

Another general challenge associated with the use of PET in clinical research is the availability and hence cost of PET tracers. Few centres around the world are able to produce ^11^C-labelled ligands, and these cannot be distributed to other hospitals because of the 20-min radioactive half-life. Moreover, production usually results in amounts of radioactivity that are sufficient for only one scanning time. The development of ^18^F-labelled ligands, that allow for scanning of a larger number of subjects for each production run as well as for transportation of tracers to other hospitals, could facilitate the more widespread and routine use of PET in clinical research. For instance, ^18^F-labelled analogues of UCB-J, such as [^18^F]SynVesT-1, have recently been developed for SV2A [43, 44]. It should be noted, however, that the longer half-life ^18^F-labelled radioligands render them unsuitable for multiple examinations during the same day, e.g., before and after an intervention, due to carry-over effects.

### PET data quantification

Besides identifying new radioligands and targets, optimizing methods for data quantification and harmonizing across centres are other areas that could accelerate the development of clinical PET research in psychiatry, as well as in other fields. For radioligands where there is no true region devoid of target, a metabolite-corrected arterial input function is required for quantification of radioligand binding, imposing limitations on clinical research in particular for vulnerable patient groups. In attempts to increase the clinical utility of TSPO PET, the use of a pseudoreference region (i.e., a region containing specific binding, hence not fulfilling the criteria for a true reference region) has been applied in several studies, however, the reliability and validity of this approach has shown to be low in healthy subjects [45, 46]. Although pseudoreference regions may prove to be useful in certain neurological conditions where the pathology can be assumed to be more restricted [47, 48], their use is more questionable in psychiatric populations where changes in immune function are expected to be subtle and diffuse. For SV2A PET a white-matter reference region has been proposed [49], and even more simplified approaches have also been evaluated [50] but competition studies showing specific binding indicate that reference tissue approaches may introduce a bias in binding estimates [51]. To ease the experimental burden, image-based input function is one area under investigation [52, 53] but the poor resolution of current PET systems, as well as head movement artefacts, limit their utility. Importantly, even if the accurate estimation of image-based input functions is achieved, apart from a few exceptions (notably [^18^F]FDG and [^15^O]water) most tracers undergo peripheral metabolism and arterial sampling for metabolite analysis will therefore still be required to assess the amount of intact tracer in blood. The possibility of using venous instead of arterial samples for metabolite analysis would result in a less invasive procedure. Although metabolite analysis based on venous sampling has been published for several tracers, direct comparison to arterial sampling has only been done in few studies with varying results [54, 55].

When full quantification using an input function is performed, the typical outcome (total distribution volume) is the sum of specific binding and non-specific and free radioligand (=non-displaceable binding). For tracers requiring this method, novel approaches to separate specific from non-displaceable binding could potentially increase sensitivity, leading to increased power in clinical studies [56, 57]. Importantly, non-displaceable binding has also shown to be a potential confounder in clinical populations, including alcohol use disorder [58]. Finally, simultaneous multifactor estimation of binding parameters using Bayesian analysis has in initial evaluations shown promise for further reducing measurement error, although the computational cost needs to be addressed for sufficient generalizability [59]. Ideally, the development of methods for quantification should be performed using open source code, to increase transparency and harmonization [60].

### PET systems

To further enhance the utility of PET in clinical studies, there is a need for new PET systems with increased sensitivity to allow for both more precise measures, increasing statistical power, and repeated measurements by means of reduced radioactive doses.

The current state of the art for brain-PET imaging, the High-resolution research tomograph (HRRT, Siemens) was designed in the early 2000s [61]. Its spatial resolution, although high, is limited by its sensitivity, i.e., the spatial resolution that can theoretically be achieved based on detector size cannot be realized in practice because of limited counts. Recent years have seen a steady increase in the axial field of view (FOV) of PET/CT systems, from a standard axial length of 15 cm about 10 years ago, to 25 or even 30 cm as standard options from most vendors [62, 63]. Since the sensitivity of PET systems is proportional to the square of their axial FOV, a factor two increase in axial FOV leads to a fourfold increase in sensitivity. More recently, total-body PET scanners have been launched, such as the uEXPLORER (United Imaging) covering the entire body with an axial FOV of 194 cm [64], or the Quadra (Siemens) head-to-thigh with an axial FOV of 106 cm [65]. In addition to an order of magnitude increase in sensitivity, these systems offer the possibility of using the left ventricle of the heart or the aorta to define image-derived input functions, and of simultaneously measuring tracer kinetics in the brain and body, allowing for studies of the brain-body connection in psychiatric disorders.

In addition to increases in sensitivity due to larger axial FOV, improved performance of time-of-flight (TOF) PET results in increases in signal to noise ratio [66]. Whereas the first commercial TOF-capable PET/CT systems from around 2010 [67] were mainly beneficial to whole-body oncological imaging due to high spatial uncertainty, the latest generation of digital PET/CT scanners has a TOF resolution of slightly over 200 ps [63], which corresponds to a spatial uncertainty of 3 cm resulting in image quality improvements also for brain measurements. Combining these lines of development, the proposed neuroEXPLORER system has an axial FOV of about 50 cm and a high TOF resolution of <250 ps with improved spatial resolution due to the reduction of detector dimensions [68]. This may result in a tenfold increase of effective sensitivity compared to the HRRT, enabling also higher spatial resolution images. The large FOV and sensitivity will for instance enable high-resolution imaging of the carotid arteries, which finally may allow for quantitatively accurate measurement of image-derived input functions from these arteries despite their small size.

The typical effective radiation dose [69] due to PET scans with ^11^C- and ^18^F-labelled ligands is around 2 mSv and between 5 and 10 mSv, respectively, limiting the possibility of performing multiple examinations in the same subjects, as the maximum effective dose in clinical research is typically limited to 10 mSv depending on the expected impact of the research. Apart from improved image quality and resolution, the development of PET systems with higher sensitivity also allows for proportional reductions in the amount of administered radioactivity. This would enable longitudinal imaging protocols with increased time resolution or studies with multiple radioligands, allowing for investigation of interactions between different molecular processes [70–72], and also the inclusion of young adults or even children.

### Multimodal approaches

In order to enhance the interpretation of findings from molecular imaging in psychiatry, a growing insight is that PET measures need to be aligned to other aspects of brain function, such as functional connectivity and neurophysiological measures (for a recent review, see [73]). Combining PET with blood-oxygenation-level dependent (BOLD) fMRI opens up for research on associations between molecular targets and neural activity in response to specific tasks. Early attempts have provided important insights into the serotonergic involvement in amygdala activity to threat-related cues [74], and more recently, advanced modelling approaches of the coupling between neurotransmitters and neural activity (fMRI) have shown how basic changes in neurotransmitter systems may affect large-scale brain network functioning and give rise to psychiatric symptoms [75]. Thus far, these studies have mainly performed PET scanning and fMRI at separate occasions, a design that has many drawbacks (e.g., different physiological states due to sleep, menstrual cycle, and food intake; and habituation or learning effects from performing the same task twice in the case of functional PET). The advent of combined PET/MR systems has enabled the simultaneous acquisition of both modalities, allowing for the exploration of more precise relations between molecular targets and hemodynamic responses. As an example, measurements of endogenous dopamine release were recently combined with fMRI to study Pavlovian fear conditioning, an experimental and translational model of anxiety disorders and PTSD [76].

As mentioned above, an obstacle to the realization of the full potential of combined fPET/fMRI is the lack of suitable radioligands for measuring changes in neurotransmitter levels during task or pharmacological challenge paradigms. In waiting for such radioligands, existing radioligands have been put to creative use. Hahn and co-workers used [^18^F]DOPA and a constant infusion design, where task-related deviation from expected linear increase in tracer uptake was calculated to study the dopaminergic underpinnings of reward processing, thereby making the temporal resolution of neurotransmitter release more equal to the level of fMRI [77]. For tracers with reversible kinetics, steady-state methods using bolus-infusion protocols can be used to measure changes in binding after a pharmacological challenge. In cases where a reference tissue is available, linear parametric neurotransmitter PET (lp-ntPET) has shown to be able to quantify and estimate the kinetics of neurotransmitter release with high temporal resolution [78].

Apart from studies on brain activation, further insight may be gained by adding functional magnetic resonance spectroscopy (fMRS) to assess neurometabolite concentrations in multimodal PET/MRS investigations. MRS is a non-invasive MR technique that exploits the change in MR signal exerted by the specific molecular surrounding of each biochemical (i.e., chemical shift) to produce a spectrum where specific peaks are associated with specific biochemicals (often called metabolites). Using MRS it is possible to quantify concentrations of a range of biochemicals including GABA, glutamate, choline, and N-acetyl-aspartate within an anatomically pre-defined brain region. However, neuroreceptors and transporters cannot be measured, and the relatively lower concentrations of, e.g., dopamine and serotonin and overlap of peaks with other more abundant chemicals hampers detection of these neurotransmitters. Moreover, most of the MRS studies to date have used single-voxel MRS with relative large voxels (>1 cm^3^) that need to be defined before data collection. MRS imaging (MRSI) can be used to produce a 2 or 3-dimensional map of spectra, but has not been applied to the same extent due to increased artefacts and longer scan times. Hence, although both PET and MRS are used to assess neurochemical processes, they differ in spatial resolution and targets. In a recent example of using MRS in conjunction with PET, measurements of prefrontal GABA with MRS and whole-brain GABA-receptor availability with [^11^C]Flumazenil PET were performed in patients with MDD compared to healthy controls [79]. A negative correlation between receptor and neurotransmitter availability was observed, which may be reflective of homoeostatic regulation. Of special interest in this context is the recent development of functional protocols to measure task-based changes in glutamate [80]. This opens up for the application of interleaved fMRS/fMRI acquisition, allowing for trimodal data collection of, e.g., glutamate and dopamine release together with hemodynamic measures of neural activity within a hybrid PET/MRI system. However, to the best of our knowledge, this approach is still to be realized.

Another emerging strategy to capture a richer picture of what molecular imaging is reflecting in terms of brain function is to combine PET with measurements of neural activity using neurophysiological techniques [79, 81]. In a recent example of this approach, the PET tracer [^11^C]Lu AE92686 was combined with both fMRI-derived striatal activity and neurophysiological striatal function measured using the mismatch negativity (MMN) response to show a link between intracellular enzyme levels, regional brain activity and behavioural brain function [81]. The combination of PET with electroencephalogram (EEG) recordings has been applied also to the serotonin system, by examining 5HT1A-R levels in relation to measures of loudness dependence of auditory evoked potentials (LDAEP) [82]. This design enabled a translation of knowledge from animal studies on the serotonergic basis of LDAEP to a clinical sample of patients with unipolar or bipolar depressive disorder. In a recent trimodal study, simultaneous EEG, molecular PET and fMRI was used to investigate functional microstates in cortical hubs in healthy participants [83]. This approach has yet to be applied to psychiatric populations. There are also other neurophysiological techniques that to the best of our knowledge so far have not been combined with molecular imaging in psychiatric samples, such as magnetoencephalography and functional near infrared spectroscopy.

### Longitudinal and interventional studies

Determining changes in imaging markers over time is an important strategy to establish causal mechanisms as well as identifying individuals with specific disease trajectories that would benefit from directed and/or early interventions. In the field of neurodegenerative disease, the identification of early stages of Alzheimer’s disease by assessing mild cognitive impairment and amyloid depositions has prompted studies investigating the association of biomarkers to disease progression [84]. Early attempts in psychiatric populations include longitudinal [^18^F]DOPA examinations in individuals with clinical high risk for psychosis, showing an increase in uptake in those individuals that converted to psychosis, and the conversion was in turn predicted by higher levels of [^18^F]DOPA at baseline [85–87]. Despite the intense focus on serotonergic markers in the field of depression and anxiety disorders, there is a lack of longitudinal studies in this area, and we are not aware of any longitudinal psychiatric PET studies that include individuals before adulthood.

An additional approach to infer causal links between molecular imaging measures and clinical symptoms is to perform measurements before and after an intervention, comparing changes in the biomarker to changes in symptoms level. Ideally, the intervention should not directly target the marker of interest. A handful of studies have assessed changes in brain neurotransmission following psychotherapy, showing increases in serotonin markers after treatment in patients with MDD [88, 89], a relationship between reductions in symptoms of social phobia and changes in D2-R binding following cognitive behavioural therapy (CBT) [90] and reduced serotonin 1B receptor (5HT1B-R) binding after CBT in patients with MDD [91]. Similarly, PET has been applied before and after electroconvulsive therapy (ECT), showing changes in binding to the 5HT1B-R as well as the dopamine transporter [92, 93] whereas no change or inconclusive results were observed in small studies investigating the 5HT1A and dopamine D2 receptors [94–96]. A recent study of how expectations may modulate treatment effects of SSRI in social anxiety disorder revealed an influence on the dopamine system as shown using PET measurements of the dopamine transporter [72].

In repetitive transcranial stimulation (rTMS) used for MDD, a variety of treatment protocols have been associated to changes in metabolic activity in remote brain areas not subjected to the actual stimulation, including frontal areas and anterior cingulate cortex (ACC) [97]. In healthy volunteers, effects of rTMS was shown on dopamine signalling as measured using [^11^C]PHNO PET [98, 99]. We could not identify any longitudinal or non-pharmacological interventional studies using radioligands for intracellular targets, immune markers or synaptic density.

### PET and precision medicine

An important step towards the application of molecular imaging in clinical practice is to find biomarkers that predict treatment response. With regard to specific molecular targets, a six-month longitudinal study in first-episode psychosis patients found that elevated [^18^F]DOPA uptake predicted a positive treatment response to antipsychotics [100]. Similarly, PET TSPO levels were recently shown to predict treatment response to celecoxib, a non-steroidal anti-inflammatory drug, in patients with the depressive disorder [101]. Earlier attempts focusing on brain metabolism include studies in obsessive-compulsive disorder where responders to paroxetine treatment showed decreased metabolism in frontal and subcortical regions [102], and a meta-analysis has suggested that metabolic activity in the ACC may have a predictive value for treatment response in geriatric depression [103]. Extending this paradigm to study treatment-specific predictors, McGrath et al. employed [^18^F]FDG PET in a sample of patients with the major depressive disorder before being randomized to either CBT or SSRI treatment. For CBT, hypometabolism in the insula (relative to the whole-brain mean) predicted remission and hypermetabolism in the same region predicted poor response, whereas the opposite pattern was noted for the SSRI escitalopram [104]. With regard to other treatment modalities, an early pilot study showed that insular [^18^F]FDG levels predicted treatment response to vagus nerve stimulation [105], and metabolism in the subgenual ACC has been observed to predict response to deep-brain stimulation (DBS) [106].

Importantly, taking the next step into true precision medicine requires evaluating the utility of a candidate biomarker for selecting treatment in prospective trials. However, these are still very scarce. Following the results by McGrath et al., the same group recently assigned treatment based on pre-treatment insular [^18^F]FDG levels, but could not provide support for insula metabolism as a treatment selection biomarker [107], and another relatively large retrospective trial including over 60 patients with MDD failed to identify any [^18^F]FDG PET derived cerebral biomarkers of citalopram or placebo response [108]. PET might also be used to identify individualized targets for brain stimulation therapies such as rTMS, DBS and transcranial focused ultrasound. However, early attempts using [^18^F]FDG PET for localization of targets for rTMS have not been successful [109, 110]. Arguably, more specific molecular targets that show a closer link to pathophysiological mechanisms are needed for treatment prediction studies, as well as the addition of multimodal functional measurements.

Once a causal mechanistic model has been established, adding relevant genetic information or blood and cerebrospinal fluid biomarkers to PET data could be a way of further enhancing the performance of prediction models. An important general aspect that needs to be taken into consideration when it comes to the use of PET in precision psychiatry is the cost-benefit trade-off including both the cost and patient radiation exposure.

#### Transdiagnostic studies

An important overarching limitation in research on the biological underpinnings of psychiatric disorders is the reliance on current diagnostic boundaries. Over a century of research has led to the insight that disturbances in human brain function and subsequent behaviour does not translate into the current diagnostic systems. As a consequence, trying to identify neurobiological determinants based on heterogeneous clinical diagnoses becomes an inefficient strategy [15], instead it is considered that the field should move towards employing transdiagnostic samples selected on more specific symptoms or behaviours. An extensive and ambitious initiative to aid researchers in this pursuit is the Research Domain Criteria (RDoC) framework [111], which consists of five domains of human behaviour that are proposed to reflect similar underlying neurobiology, with several dimensions that would allow the investigation of disease pathways from genes, to molecules, circuits and symptoms. This brings clear theoretical advantages for translational studies, but thus far no consensus exists on how to specifically assess these domains in clinical samples. Moreover, since the criteria were launched in 2011 only a minority of the first 48 published proposals of RDoC-related studies included more than one diagnostic category in the study sample, and only 10 examined more than one domain [112]. To date, there are only a few PET studies fully embracing the RDoC-strategy. Langenecker et al. studied patients with MDD, finding that lowered 5HT1A-R binding corresponded to a subtype with the decreased engagement of the cognitive control network and impaired resolution of interfering cognitive stimuli [113].

### Approaches to increase sample size

Another general limitation in psychiatric PET research, as in other areas of molecular imaging, is the use of small samples. Notably, this gives rise not only to low sensitivity for true effects but also increases the risk for type II errors [114]. Given the high costs and sometimes cumbersome research protocols, data sharing across centres is an important way to increase sample sizes. For instance, in the field of TSPO, data from 140 healthy individuals examined using [^11^C]PBR28 was pooled to show the effects of gender, age and body mass index which had not been conclusively demonstrated before [115]. Similarly, employing an individual participant data metanalysis approach, Plavén-Sigray et al. synthesized TSPO data from psychosis and schizophrenia data collected at six different centres, using three different second-generation radioligands. The best fit was obtained was for the statistical model where differences in standardized binding did not vary between centres, indicating that methodological differences in data collection were not a key factor [23, 116].

In order to examine both within-diagnosis heterogeneity as well as cross-diagnostic research questions, combining samples becomes even more essential. To facilitate this development, it would of great value to collect data on dimensional symptoms, to allow for diagnostic-agnostic investigations of the biological basis for symptoms, across samples. Similarly, it is advised to include in the informed consent that data may be shared as part of international collaborations [117]. Data sharing has since long been employed in the field of dementia in the form of the Alzheimer’s Disease Neuroimaging Initiative, generating more than 3500 publications [118], as well as for MR imaging and genetics in psychiatry through the ENIGMA consortium [119]. In the field of PET, harmonized protocols for data acquisition, pre-processing and data structure, for instance, using the Brain Imaging Data Structure (BIDS) specifications could additionally facilitate data sharing [120].

### Ethical issues

Another challenge when performing molecular imaging in psychiatry is the population of severely ill patients who may not be able to provide informed consent to research participation. An example is catatonia, a striking clinical presentation of a severe psychomotor disturbance, which can occur across diagnostic categories. It has been hypothesized that the symptom may have distinct neurobiological underpinnings, based on the dramatic effects of interventions such as lorazepam injection or ECT [121]. Yet, our understanding of the neurobiology of catatonia is precluded by the lack of systematic large-scale studies. Case reports reporting molecular imaging findings have pointed to a frontal-parietal hypofunction [122], however, all these studies investigated patients only after having been successfully treated with lorazepam. In one of the few studies using informed consent from peers instead of from the patients, patients were investigated during the actual catatonic state before and after ECT. In this study another pattern emerged, with increased blood flow in parietal, temporal, and occipital regions [123], suggesting that examining patients during the actual state can provide more relevant information. Difficulties of investigating severe illnesses in patients who cannot provide informed consent are shared with other areas of research such as emergency research and dementia studies, where there are recommendations available on situations where a consent waiver could be applied for intervention studies [124].

Other areas where ethical issues can be present are studies of paediatric populations or young individuals with prodromal signs of psychotic illness. Studies using molecular imaging early in the development of a brain disorder can be critical for the understanding of key disease mechanisms and how they may be modified or even prevented. An example of this is studies of autism during childhood and adolescence where an aberrant development of the serotonergic system has been revealed, suggesting new possible interventions [125]. Hence, whereas it could be considered ethical to refrain from studying individuals who cannot provide informed consent from an individual level, it could at the same time be considered unethical not to study the disorder from a patient population level.

## Conclusions and future directions

**Fig. 2:**
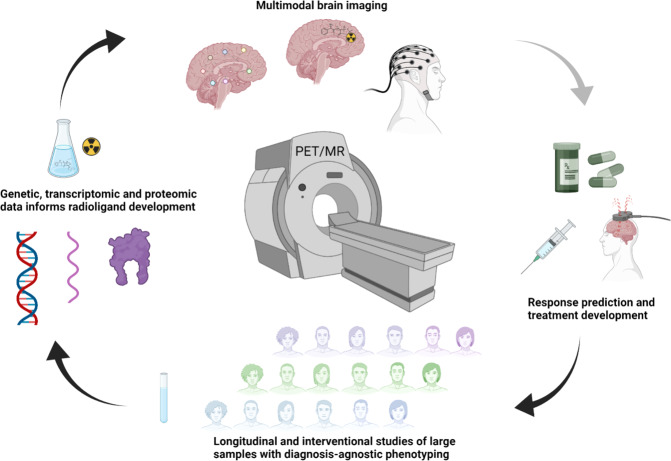
Summary of proposed areas of development in psychiatric PET research. Figure created with BioRender.com.

The contribution of mental disorders to the global disease burden has increased from 3.1 to 4.9% between 1990 and 2019, corresponding to 125.3 million disability-adjusted life-years (DALYs) [126]. This disability is distributed across all age groups, with a peak between 25 and 34 years. Notably, these estimates did not include substance use disorders which together account for similar proportions of disability [127]. For Alzheimer’s disease, a brain disorder causing approximately 5.2 million DALYs [128], the estimated research budget for the National Institute of Health in the US for 2022 was 3.2 billion USD, whereas for all mental disorders (except substance use disorders) the corresponding amount was 1.7 billion USD [129]. Although not necessarily reflecting global priorities, these figures indicate that an increased focus on research on mental disorders is highly warranted, including investing in the development of methods specifically designed for this purpose (Fig. 2).

The discovery and marketing of antipsychotics, acting mainly on the dopamine system, in the 1950s and serotonergic antidepressants in the 1980s, was the main basis for two major disease models in psychiatry – the dopamine hypothesis of schizophrenia, and the serotonin hypothesis of depression. These conceptualizations have been a powerful driver of research into pathophysiology, resulting in a strong focus of psychiatric molecular imaging research on monoaminergic brain neurotransmission systems. The lack of resulting diagnostic tools or new treatment approaches indicate that causal mechanisms may involve other aspects of brain function, hence identifying new targets and radioligands is a critical step to advance psychiatric PET research. A concern is that programs for radioligand development are often driven by the pharmaceutical industry to validate compounds based on existing disease models, such as those above, or aimed towards other disease areas such as neurodegeneration or oncology, rather than being informed by new hypotheses regarding psychiatric disorders. The immune system has been implicated in both genetic and biomolecular studies across several psychiatric diagnoses [24], and with regard to schizophrenia, recent genetic and experimental data have suggested a mechanistic model involving immune-neuronal interactions, converging on aberrant synaptic development and plasticity [130–132]. These new disease models have indeed led to a shift of focus, and new radioligands for immune markers and synaptic density have to some extent been applied in psychiatric conditions, but much remains unexplored. For instance, we could not identify any longitudinal, intervention or treatment prediction studies involving these radioligands.

Psychiatric conditions in general are likely not caused by loss of neurons or tissue damage. Instead, symptoms are thought to reflect changes in dynamic brain function and plasticity, leading to aberrant information processing. Hence, these aspects need to be incorporated in order to fully understand the results of molecular imaging studies. A growing number of studies are combining PET with other imaging modalities such as fMRI as well as neurophysiological measures. These paradigms have mainly targeted monoaminergic brain neurotransmission systems, and should now also be extended to new putative markers of pathophysiology. In addition, there is increasing evidence indicating that psychiatric symptoms may be a result of an interplay between the brain and other organs such as the gut [133], suggesting that molecular imaging studies in some cases could be extended to include also whole-body examinations.

Whereas some psychiatric conditions are by definition considered neurodevelopmental disorders (autism spectrum disorders and attention-deficit/hyperactivity disorder), an emerging view also for other diagnoses is that genetic and environmental risk factors interact over longer time periods before symptoms present (e.g. refs. [134, 135]). Hence, for many conditions, investigations may need to be initiated already during childhood or adolescence to fully capture the entire disease course. One important aspect of methodological development is to increase sensitivity such that radioactivity dosing can be reduced, allowing both for repeated examinations and studies in early life, as well as investigating multiple targets in the same individual. Moreover, disease trajectories are often heterogeneous, spanning from continuous symptom presentation and disability to relapsing-remitting courses. There is a clear lack of molecular imaging studies in psychiatric patients over longer time periods, for instance comparing periods of active disease to periods of higher functioning. Again, an important requisite for this development is a new methodology to allow for the administration of lower amounts of radioactivity.

Examining individuals with severe mental disorders involves both practical and ethical challenges. In order to gain information regarding these conditions, it is vital to simplify experimental procedures as well as limit the number of research visits. Methods to quantify radioligand binding without the use of an arterial input function, methodology allowing for shorter times of acquisition, as well as simultaneous multimodal measurements are important steps in this direction.

The definition of psychiatric diagnoses has essentially remained the same since before the advent of genetic research and modern neuroscience. Despite that psychiatric diagnoses are generally acknowledged to not reflect underlying biology, most research using molecular imaging have aimed to compare specific diagnostic populations to unaffected individuals. Initiatives such as the RDoC [111] have been applied to a very limited extent in psychiatric PET research, limiting the disentangling of specific symptom domains from the imposed diagnostic framework. Ideally, researchers should strive to include both diagnostic information and assessment of dimensional symptoms in studies. This would allow both for the identification of subgroups within diagnoses and common biological aberrations across disorders.

A general constraint in PET research has been small samples, both limiting the inferential value of results and hampering research into diagnostic heterogeneity. In a field with limited resources, systems for grant distribution and publication lead to a focus on novelty rather than broad, systematic research programs. At present, in order to reach desired sample sizes, collaboration and pooling across centres are necessary. Initiatives to harmonize research protocols and data acquisition is an important development towards this goal. This would also require a shift of focus in grant providers, as well as legislators regarding the possibility of sharing information across regional and national boundaries, such that researchers can engage in a concerted action to diminish the global burden of mental disorders.

In conclusion, by being the only method that can directly access brain molecular processes we believe that PET will play an increasingly important part in understanding the aetiology, progression, and treatment of the highly impairing psychiatric disorders. However, this requires substantial investments to accelerate the development across several areas, including identifying and validating suitable radioligands, more precise acquisition and quantification methods, multimodal imaging protocols, longitudinal and interventional paradigms in large samples, as well as data sharing and collaboration.
